# Impact of Implant Size and Position on Subsidence Degree after Anterior Cervical Discectomy and Fusion: Radiological and Clinical Analysis

**DOI:** 10.3390/jcm13041151

**Published:** 2024-02-18

**Authors:** Adam Bębenek, Maciej Dominiak, Grzegorz Karpiński, Tomasz Pawełczyk, Bartosz Godlewski

**Affiliations:** 1 Department of Orthopaedics and Traumatology, with Spinal Surgery Ward, Scanmed—St. Raphael Hospital, 30693 Cracow, Poland; 2 Department of Affective and Psychotic Disorders, Medical University of Lodz, 92216 Lodz, Poland; tomasz.pawelczyk@gmail.com

**Keywords:** subsidence, degenerative, disc, cervical, spine, spondylodesis, intervertebral, ACDF

## Abstract

Background: Implant subsidence is recognized as a complication of interbody stabilization, although its relevance remains ambiguous, particularly in terms of relating the effect of the position and depth of subsidence on the clinical outcome of the procedure. This study aimed to evaluate how implant positioning and size influence the incidence and degree of subsidence and to examine their implications for clinical outcomes. Methods: An observational study of 94 patients (157 levels) who underwent ACDF was conducted. Radiological parameters (implant position, implant height, vertebral body height, segmental height and intervertebral height) were assessed. Clinical outcomes were evaluated using the Visual Analogue Scale (VAS) and Neck Disability Index (NDI). Subsidence was evaluated in groups according to its degree, and statistical analyses were performed. Results: The findings revealed that implant-to-endplate ratio and implant height were significant risk factors associated with the incidence and degree of subsidence. The incidence of subsidence varied as follows: 34 cases (41.5%) exhibited displacement of the implant into the adjacent endplate by 2–3 mm, 32 cases (39%) by 3–4 mm, 16 cases (19.5%) by ≥4 mm and 75 (47.8%) cases exhibited no subsidence. Conclusions: The findings underscore that oversized or undersized implants relative to the disc space or endplate length elevate the risk and severity of subsidence.

## 1. Introduction

Anterior cervical discectomy and fusion (ACDF) is a commonly employed surgical intervention for cervical spondylopathy, utilizing the Smith–Robbinson approach. The procedure entails two key stages, namely decompression and implantation. During the decompression phase, the operator removes any material that may cause pressure, irritate nerve roots, or affect the spinal cord from the intervertebral space [1]. Through the strategic implantation of material into the affected disc space, we are able to generate the requisite conditions for fusion to take place. This process stabilizes the impacted segment and effectively mitigates the potential for disease progression [2,3,4]. Recent advancements in materials technology have led to a shift away from the traditional use of iliac grafts in medical procedures. Due to the complications associated with harvesting these grafts, allogenic alternatives are now being used instead [4,5]. The ultimate objective of ACDF is to achieve a successful bone fusion at the operated level, which is crucial for the therapy’s effectiveness. Unfortunately, complications may arise for various reasons. Some of these complications may be directly related to the surgical procedure, such as dysphagia, postoperative bleeding, CSF leakage, discomfort, or Horner’s syndrome. Additionally, further complications may arise over time as a result of graft placement. Key among them are subsidence, pseudarthrosis and cervical malalignment [6,7]. The above-mentioned complications are interrelated. Theoretically, the subsidence phenomenon can cause cervical malalignment, which, as a result of non-physiological biomechanical loads on the segment, increases the risk of pseudoarthrosis. Pseudoarthrosis, in turn, causes instability, which exacerbates the aforementioned non-physiological loads manifested by increasing osteochondral changes that can aggravate the clinical manifestations of spondylopathy [7,8]. Numerous researchers have invested considerable effort and resources to expand our understanding of subsidence, recognizing its importance. Noordhoek et al. conducted a comprehensive analysis in 2018, revealing that subsidence occurs on average in 20.2% of cases, with a range of 0% to 83%. However, the impact on clinical outcomes remains uncertain due to the varying results and the potential for bias in the studies examined [7]. Different authors have varying definitions for subsidence, leading to inconsistent criteria based on absolute values or numerical ratios [8,9,10,11,12,13]. To assess the operated level and compare preoperative and postoperative states, X-rays are used. They enable the monitoring of bone fusion and the detection of subsidence. In this article, the authors put forth a hypothesis suggesting a potential correlation between the depth of subsidence and clinical outcomes. Moreover, they undertake an investigation into the potential impact of radiological parameters pertaining to implant dimensions and positioning on the incidence and degree of subsidence.

## 2. Materials and Methods

### 2.1. Study Design

**Figure 1:**
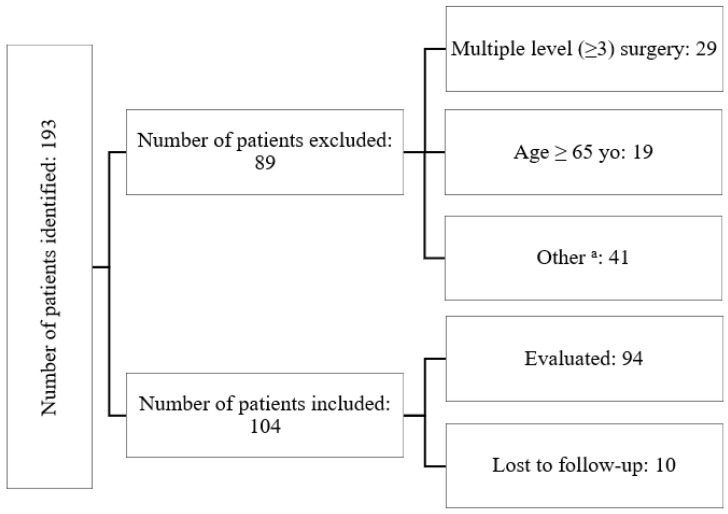
The chart introducing the patients flow throughout the study; ^a^—the “other” group encompassed patients who had active rheumatologic/metabolic diseases or had previously undergone surgery at a different level.

The observational study was conducted exclusively at a single center, involving a cohort of 104 patients who underwent anterior cervical discectomy and fusion (ACDF) surgery for cervical disc disease spanning the period from 2019 to 2021. Patients eligible for inclusion exhibited diagnosed cervical degenerative disc disease that was unresponsive to conservative treatment, confirmed via preoperative MRI scans. Inclusion criteria stipulated an age range between 18 and 65 years and suitability for either single- or double-level ACDF surgery. The exclusion criteria encompassed individuals outside the age range (older than 65 years or younger than 18 years), those with concurrent osteoporosis or active rheumatologic/metabolic diseases, prior surgical intervention at a different level, and patients necessitating three or more levels of surgical intervention. Among the 193 individuals assessed during the study timeframe, 104 fulfilled the predetermined inclusion criteria (Figure 1).

### 2.2. Procedure and Implants

All surgeries adhered to the standardized Smith–Robbinson approach. The intervertebral implants utilized in all cases shared identical dimensions, measuring 11.5 mm in length and 14 mm in width, ensuring uniform surface area across all implants. Variations were only observed in terms of height and material composition. Specifically, the study included 85 implants composed of poly-ether-ether-ketone (PEEK) and 72 titanium-coated PEEK (TC) implants sourced from a single manufacturer (Aesculap Chifa, CeSPACE^®^ Implants, Tuttlingen, Germany). These implants offered a height range of 4–8 mm, with a mean of 6.3 mm and a median of 6 mm. Each implant featured a nanoparticle hydroxyapatite filler obtained from the same manufacturer (B Braun, Nanogel^®^ Hydroxyapatite, Melsungen, Germany). Notably, stand-alone cages were exclusively used, and plating was omitted from the procedures.

### 2.3. Radiological Assessment, Clinical Evaluation and Subsidence Criteria

**Figure 2:**
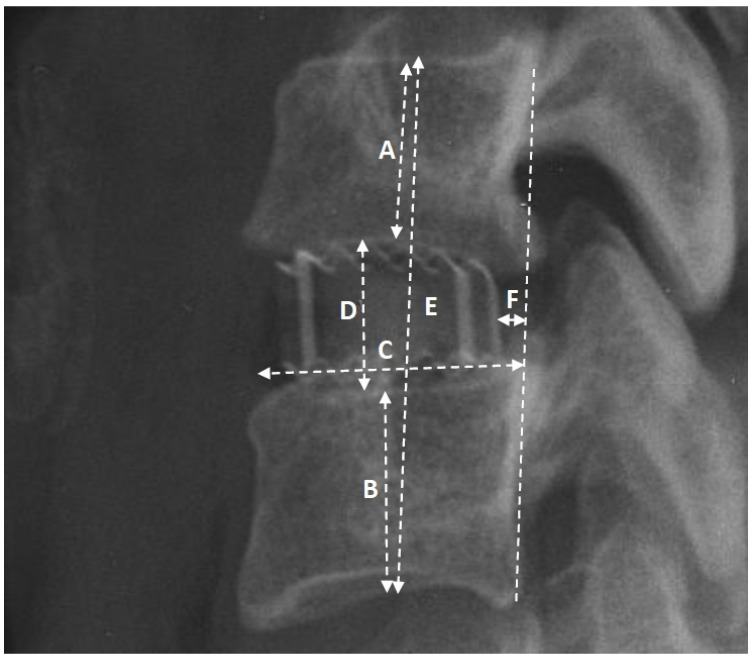
The image of the operated segment in the lateral projection, showcasing the selected radiological parameters utilized in the study: A—height of the upper body; B—height of the lower body; C—length of the lower endplate; D—height of the intervertebral space; E—height of the segment; F—distance of the implant from the medial column (posterior longitudinal ligament).

**Figure 3:**
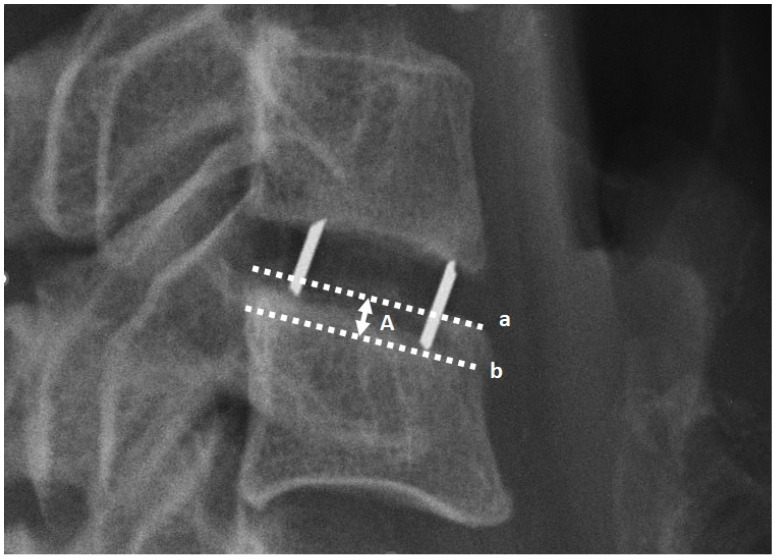
The image illustrates the subsidence measurement method based on the displacement of the cage into the adjacent endplate. ‘A’ represents the depth of cage displacement, ‘a’ refers to the line tangent to the endplate, and ‘b’ represents the line tangent to a passing through the lowest point of the cage.

Radiological assessments were conducted via X-rays in lateral projection at five distinct intervals: (1) the day preceding the surgery, (2) the immediate postoperative day, (3) one month post-surgery, (4) six months post-surgery, and (5) twelve months post-surgery. All radiographic images were acquired at the authors’ facility, employing consistent equipment and adhering to standardized procedures. Measurements were meticulously obtained with a precision of 0.1 mm. The assessed radiological parameters, illustrated in Figure 2, underwent evaluation. Clinical outcomes were appraised using the Visual Analogue Scale (VAS) and Neck Disability Index (NDI) during the follow-up imaging sessions. Subsidence was recognized in case implant displacement into the adjacent endplate by ≥2 mm. To assess the degree of subsidence, 3 categories were created based on the magnitude of implant displacement compared to the radiographs taken the day after surgery: (1) implant displacement into the adjacent endplate by ≥2 mm and <3 mm, (2) implant displacement into the adjacent endplate by ≥3 mm and <4 mm, and (3) implant displacement into the adjacent endplate by ≥4 mm. The measurement method is depicted in Figure 3.

### 2.4. Statistical Analysis

The comparison of quantitative variables between the groups was performed using either the Mann–Whitney test, Student’s *t*-test for independent variables or the Welch T-test. In the case of an analysis of more than two groups, Kruskal–Wallis or ANOVA tests were performed. The analysis utilized a significance level of 0.05, wherein *p*-values below 0.05 were deemed to signify significant relationships. Statistical computations were performed using MedCalc^®^ Statistical Software version 20.104 (MedCal Software Ltd., Ostend, Belgium) and TIBCO Statistica 13.3 (TIBCO Software Inc., Palo Alto, CA, USA). The study received approval from the Bioethics Committee of the Andrzej Frycz Modrzewski University in Cracow (Resolution 4/2019—24 January 2019) and adhered to the principles outlined in the Declaration of Helsinki. All patients and/or their legal guardian(s) gave their written consent, were informed about the purpose and conduct of the study, and knew that the data acquired would be submitted for publication.

## 3. Results

### 3.1. Participants

Among the cohort of 104 eligible patients, comprising 76 women and 28 men, the mean age was 51 years, with a median age of 50 years. During the 12-month follow-up period, 10 patients were lost to follow-up, resulting in the evaluation of images from 94 patients (representing 157 intervertebral spaces). Among these, 31 cases involved a single level, while 63 cases involved double levels. Over the course of the 12-month period, there was an average clinical improvement of 3.6 points in the Visual Analogue Scale (VAS) scores and 14 points in the Neck Disability Index (NDI) scores. No statistically significant difference was observed in terms of gender, age, type of implant and number of levels instrumented (*p* value > 0.05) (Table 1).

### 3.2. Subsidence and Implant Placement

Regarding the segment height (E), the average values were as follows: 36.4 mm before surgery, 38.6 mm the day after surgery, 37.3 mm after one month, and 36.5 mm and 36.1 mm after 6 and 12 months, respectively. The height of the intervertebral space (D) exhibited the following averages: 5.7 mm before surgery, 8.6 mm the day after surgery, 7.9 mm after one month, and 7.1 mm and 6.7 mm after 6 and 12 months, respectively. The mean height of the implant used was 6.16 mm, with a median of 6 mm. Additionally, the average length of the endplate (C) was 21.5 mm, while the mean distance of the implant from the medial column (F) was 4.2 mm. Furthermore, the mean heights of the upper (A) and lower (B) vertebral bodies were 15.4 mm and 15.8 mm, respectively. In terms of subsidence, within the study group of 157 intervertebral spaces, the frequencies were as follows: 82 cases (52.2%) exhibited displacement of the implant into the adjacent endplate by ≥2 mm, whereas 34 cases (41.5%) exhibited displacement of the implant into the adjacent endplate ≥2 and <3 mm, 32 cases (39%) exhibited displacement of the implant into the adjacent endplate by ≥3 and <4 mm, 16 cases (19.5%) exhibited displacement of the implant into the adjacent endplate by ≥4 mm, and 75 (47.8%) cases exhibited no subsidence. The average ratio of the distance of the cage from the middle column divided by the length of the endplate. (F/C on Figure 2) was 0.19. However, this ratio did neither exhibit statistical significance as a factor influencing occurrence nor the degree of subsidence (Table 2). Another statistical analysis was conducted to assess the relationship between the cage-to-endplate length ratio and its contribution to subsidence. The average cage-to-endplate ratio was 0.52, and it was found to be statistically significant for subsidence: 0.51 vs. 0.53 with *p* = 0.0448. However, no significant relationship was observed for the degree of subsidence (Table 2). Furthermore, when the aforementioned ratio was divided by the cage distance and referred to as the cage–endplate distance ratio, it demonstrated no statistical significance for subsidence: 0.22 vs. 0.17 (*p* = 0.1273) and no significant differences were found for subsidence degree. The ratio of implant height to intervertebral space height before surgery averaged 0.94 and exhibited statistical significance as a factor affecting subsidence: 1.18 vs. 1.1 (*p* = 0.0367), with a greater ratio for the subsidence group. Moreover, this ratio was found to be a statistically significant factor influencing the degree of subsidence with means of 1.1, 1.16 and 1.3 for groups of ≥2 and <3 [mm], ≥3 and <4 [mm] and ≥4 mm, respectively (*p* = 0.0447).

Similarly, the ratio of implant height to segmental height before surgery averaged 0.17 and demonstrated statistical significance as a factor influencing subsidence occurrence (*p* = 0.0322) and its degree (*p* = 0.0389). Additionally, the ratio of vertebral body height before surgery to implant height was also analyzed and evaluated for its impact on both subsidence incidence rate and depth. Both the above and below vertebrae had a ratio of 0.4 and did not prove to be statistically significant as a risk factor for subsidence (Table 2).

### 3.3. Subsidence and Clinical Outcome

Following a 12-month postoperative assessment, VAS and NDI scores were also evaluated in two distinct groups: one with subsidence and the other without. In the subgroup with subsidence, the mean VAS score registered was 2.3, whereas without subsidence recorded a mean VAS score of 2.0. Nevertheless, this disparity did not achieve statistical significance (*p* = 0.1123). Conversely, a substantial discrepancy was observed in the NDI scores (*p* = 0.0105) between the two cohorts, with the subsidence group presenting a mean NDI score of 12.4 and the group without subsidence exhibiting a mean NDI score of 8.5 points (Table 3). To examine the association between clinical outcomes and the degree of subsidence, a statistical analysis of VAS and NDI scores was conducted within subgroups categorized by varying depths of subsidence. The findings revealed that there was no statistically significant difference in the VAS scores relative to the depth of subsidence (*p* = 0.4733). However, a significant difference emerged with respect to the NDI scores, which exhibited an increasing trend in values corresponding to deeper levels of subsidence (*p* = 0.0459) with a mean of 10.7 pts for the 2–3 mm subgroup, 11.9 pts for 3–4 mm and 13.5 pts for ≥4 mm of implant migration into adjacent endplate (Table 3).

## 4. Discussion

Since the mid-20th century, ACDF has been the established treatment for cervical degenerative disc disease. Initial approaches to intervertebral space fusion using autograft from the iliac plate resulted in a high incidence of local complications, leading researchers to explore implants made from alternative materials [3,7,9]. Presently, commonly used cages are composed of materials such as stainless steel, titanium, carbon fiber, polymethyl–methacrylate (PMMA), and polyether–ether–ketone (PEEK) [7]. While artificial cages are designed to theoretically preserve lordosis, restore intervertebral space height, and promote bony fusion through enhanced osteointegration, complications such as fusion failure, kyphotic malalignment of the cervical spine, and subsidence can still arise [14]. This study focuses on the phenomenon of subsidence, investigating its radiological risk factors and the clinical implications based on the degree of implant migration into adjacent endplates.

Subsidence is a frequently encountered phenomenon; however, its precise definition, particularly concerning the specific depth of this occurrence, remains a subject of uncertainty and ambiguity in certain aspects [8,15]. Karikari et al. presented fundamental variations in the definition of subsidence in their 2014 article, evaluating the impact of different subsidence depth measures on radiological and clinical outcomes through a systematic review. Their work consolidates various approaches to assess subsidence, incorporating diverse cutoff points, at times interpreted as absolute values and occasionally as ratios [15]. Karikari et al. center their study on identifying risk factors for subsidence and evaluating pseudoartosis and fusion based on definitions established by previous researchers. They underscore the necessity for research geared towards establishing precise cutoff values for subsidence depth. Nevertheless, their investigation does not encompass a comprehensive analysis of how the depth of subsidence influences the clinical outcomes of the procedure [15]. In this study, our specific focus centers on the precise examination of the relationship between subsidence degree and clinical outcomes, taking into consideration various aspects of implant size and positioning.

During the procedure, the operator can choose to place the implant closer to the anterior or posterior surface of the vertebral body, where it is in proximity to the respective cortical bone, forming the anterior or posterior wall. Alternatively, the implant can be positioned more centrally without additional support. Previous authors have addressed this topic, suggesting a relationship between the distance of the implant from the anterior surface of the vertebral body and the occurrence of subsidence, as well as a correlation between the ratio of implant length to endplate length and subsidence [8,16,17,18]. The findings of this study confirm such relationships in terms of implant length to endplate length ratio. A lower incidence of subsidence was noted when the ratio of implant length to endplate length was higher: 0.50 vs. 0.53 (*p* = 0.0448). Regarding the cage–endplate to distance ratio, which reflects the positioning of the implant within the intervertebral space, no statistically significant differences were observed. It is advisable to strive for maximum overlap between the implant length and the endplate length in order to minimize the risk of subsidence.

The appropriate matching of implant height to segmental or intervertebral space height has also been considered by other authors [7,17,19]. Implants with greater height may exert more pressure on adjacent endplates than their smaller counterparts, potentially increasing the incidence of subsidence [7,17]. In 2007, Barsa and Suchomel investigated whether the degree of distraction, defined as the ratio of preoperative to postoperative disc space height, has an impact on subsidence. However, their findings at that time did not yield statistically significant results [19]. In a 2012 publication, Yamagata et al. reported a higher incidence of subsidence with 6.5 or 7.5 mm implants compared to 4.5 and 5.5 mm implants. They defined subsidence as the implant being recessed into the adjacent lamina by at least one-third of its height. Considering the range of implants used (4.5–7.5 mm), it can be inferred that the observed subsidence corresponded to a recess of approximately 1.5–2.5 mm. Additionally, their study revealed a stronger association between the ratio of implant height to segment height and the occurrence of subsidence [17]. Consistent results were obtained in our study. In the case of subsidence incidence, we obtained a statistically significant difference for both the cage-to-segmental height ratio (*p* = 0.0322) and cage-to-preoperative intervertebral space height ratio (*p* = 0.0367). In addition, a decrease in the values of the described ratios was observed as the degree of subsidence increased (Table 2). Therefore, it is reasonable to assume that the selection of an oversized implant affects subsidence by creating too much stress on the endplates of the fused vertebral bodies. Moreover, the greater the load, the greater the degree of subsidence. Precisely sizing implants to match the intervertebral space is crucial in preventing subsidence.

The heterogeneity described at the beginning of the discussion is also evident in the clinical significance of subsidence. Indeed, there is a discrepancy in the results concerning the relationship between subsidence and the patient’s clinical condition [7]. Studies by Kast et al., Lee et al., and Kim et al. report the presence of an association between subsidence and worse clinical outcomes [20,21,22]. However, numerous reports suggest the absence of such a relationship [7,23,24,25]. In our study, we assessed the VAS and NDI scale scores following a 12-month follow-up period and examined their relationship with the incidence and degree of subsidence. All patients demonstrated improvement, with average score reductions of 3.6 points on the VAS scale and 13.64 points on the NDI scale (Table 1). Furthermore, the NDI score after 12 months exhibited a statistically significant difference between the groups with and without subsidence with *p* = 0.0105 (Table 2). Additionally, we observed an increase in mean NDI scale values as the degree of subsidence escalated (*p* = 0.0459). Hence, it is reasonable to infer that both the occurrence and degree of subsidence may indeed influence the clinical outcome of the procedure.

## 5. Conclusions

The occurrence of subsidence continues to pose uncertainties regarding its impact on the outcomes of surgical interventions for spinal degenerative disease. While discrepancies exist in the impact of implant dimensions and positioning on the likelihood of subsidence, these variables merit meticulous consideration during surgical strategizing. Implants that exceed appropriate dimensions relative to the disc space or inadequately match the length of the border plate may escalate the susceptibility to subsidence and intensify its severity. Furthermore, the findings of this study suggest a conservative assumption that subsidence impacts the clinical efficacy of treatment, implying a potential inverse correlation between the degree of subsidence and treatment outcomes.

## Figures and Tables

**Table 1:** Characteristics of the study population (*n* = 94).

Characteristics	Value
Age, year, mean (range)	50 (31–65)
Gender: female, *n* (%)	67 (71.3%)
Type of implant:	
PEEK, *n* (%)	85 (54.1%)
TC-PEEK, *n* (%)	72 (45.9%)
Type of spinal fusion:	
Single-level, *n* (%)	31 (33%)
Double-level, *n* (%)	63 (67%)
C3/C4, *n*	2 (2.3%)
C4/C5, *n*	0 (0%)
C5/C6, *n*	27 (28.9%)
C6/C7, *n*	2 (2.3%)
C3–C5, *n*	4 (4.5%)
C4–C6, *n*	15 (16.1%)
C5–C7, *n*	43 (45.9%)
VAS, pts, mean:	
Preoperative	5.9
1 month after the surgery	2.4
6 months after the surgery	2.2
12 months after the surgery	2.2
∆VAS	3.6
NDI, pts, mean:	
Preoperative	24
1 month after the surgery	14
6 months after the surgery	11
12 months after the surgery	10
∆NDI	14

**Table 2:** Dependence of the occurrence and degree of subsidence on selected radiological ratios. a—statistical analysis using Mann–Whitney test; b—statistical analysis using independent *t*-test; c—statistical analysis using Welch test; d—Kruskal–Wallis test; e—univariate ANOVA; 1—the ratio between the length of the cage and the length of the endplate (11.5/C in Figure 2); 2—the quotient obtained by dividing the ratio of cage length to endplate length by the distance of the cage from the middle column [(11.5/C)/F in Figure 2]; 3—the ratio was calculated by dividing the distance of the cage from the middle column by the length of the endplate (F/C in Figure 2); 4—the ratio between the height of the implant and the preoperative intervertebral space (implant height/D in Figure 2); 5—the ratio between the height of the implant and the preoperative segmental height (implant height/E in Figure 2); 6—the respective ratio between the implant height and the upper vertebral body height (implant height/A in Figure 2) and lower vertebral body height (implant height/B in Figure 2). Statistically significant results in bold.

		Subsidence		Subsidence Degree		
		Yes	No	≥2 and <3 [mm]	≥3 and <4 [mm]	≥4 [mm]
	Number of disc spaces: (%)	82 (52.2%)	75 (47.8%)	34 (41.5%)	32 (39%)	16 (19.5%)
Parameters						
Cage-to-endplate length ratio ^1^	Mean:	**0.51**	**0.53**	0.52	0.51	0.50
	Coefficient:	**T = 1.79 ^b^**		H = 1.04 ^d^		
	*p* value	**0.0448 ^b^**		0.5947 ^d^		
Cage–endplate distance ratio ^2^	Mean:	0.22	0.17	0.29	0.18	0.15
	Coefficient:	Z = 1.53 ^a^		H = 2.79 ^d^		
	*p* value	0.1273 ^a^		0.2477 ^d^		
Cage distance to endplate length ratio ^3^	Mean:	0.20	0.19	0.22	0.19	0.17
	Coefficient:	Z = −0.92 ^a^		H = 2.24 ^d^		
	*p* value	0.3602 ^a^		0.3255 ^d^		
Cage-to-preoperative intervertebral space height ratio ^4^	Mean:	**1.18**	**1.1**	**1.1**	**1.16**	**1.3**
	Coefficient:	**Z = 2.14 ^a^**		**H = 4.69 ^d^**		
	*p* value	**0.0367 ^a^**		**0.0447 ^d^**		
Cage-to-preoperative segmental height ratio ^5^	Mean:	**0.18**	**0.16**	**0.16**	**0.18**	**0.2**
	Coefficient:	**−1.53 ^c^**		**F = 2.54 ^e^**		
	*p* value	**0.0322 ^c^**		**0.0389 ^e^**		
Cage to upper vertebral body height ratio ^6^	Mean:	0.41	0.40	0.41	0.42	0.40
	Coefficient:	T = −0.54 ^b^		F = 0.32 ^e^		
	*p* value	0.5893 ^b^		0.7237 ^e^		
Cage-to-lower vertebral body height ratio ^6^	Mean:	0.40	0.40	0.40	0.41	0.37
	Coefficient:	Z = 0.27		F = 2.27 ^e^		
	*p* value	0.7855 ^a^		0.1100 ^e^		

**Table 3:** Dependence of the Visual Analogue Scale (VAS) and Neck Disability Index (NDI) values on degree of subsidence. a—statistical analysis using Mann–Whitney test; b—statistical analysis using Kruskal–Wallis test. Statistically significant results in bold.

Subsidence		VAS Score after 12 Months				NDI Score after 12 Months			
	*n* (%)	Mean [pts]	Median [pts]	Z Coefficient ^a^	*p* Value ^a^	Mean [pts]	Median [pts]	Z Coefficient ^a^	*p* Value ^a^
**Y**	82 (52.2%)	2.3	2.0	−1.59	0.1123	**12.4**	**9.0**	**−2.56**	**0.0105**
**N**	75 (47.8%)	2.0	1.5			**8.51**	**7.0**		
**Subsidence Degree [mm], *n***		**Mean [pts]**		H Coefficient ^b^	***p* Value ^a^**	**Mean [pts]**		**H Coefficient ^b^**	***p* Value ^b^**
**≥2 and <3**	34	1.9		1.52	0.4733	**10.7**		**7.84**	**0.0459**
**≥3 and <4**	32	2.2				**11.9**			
**≥4**	16	2.5				**13.5**			

## Data Availability

The datasets analyzed during the current study are not publicly available due to legal constraints but are available from the corresponding author upon reasonable request.
